# The Role of Galectins in Asthma Pathophysiology: A Comprehensive Review

**DOI:** 10.3390/cimb46050260

**Published:** 2024-05-03

**Authors:** Andrea Portacci, Ilaria Iorillo, Leonardo Maselli, Monica Amendolara, Vitaliano Nicola Quaranta, Silvano Dragonieri, Giovanna Elisiana Carpagnano

**Affiliations:** 1 Institute of Respiratory Disease, Department of Translational Biomedicine and Neuroscience, University “Aldo Moro”, 70121 Bari, Italy; iorillo.ilaria@gmail.com (I.I.); jaleo.maselli@gmail.com (L.M.); monica.amendolara91@gmail.com (M.A.); silvano.dragonieri@uniba.it (S.D.); elisiana.carpagnano@uniba.it (G.E.C.); 2 Respiratory Disease Department, “Di Venere” Hospital, 70121 Bari, Italy; vitalianonicola.40@gmail.com

**Keywords:** galectin, asthma, Th2 inflammation, eosinophils, airway hyperresponsiveness

## Abstract

Galectins are a group of β-galactoside-binding proteins with several roles in immune response, cellular adhesion, and inflammation development. Current evidence suggest that these proteins could play a crucial role in many respiratory diseases such as pulmonary fibrosis, lung cancer, and respiratory infections. From this standpoint, an increasing body of evidence have recognized galectins as potential biomarkers involved in several aspects of asthma pathophysiology. Among them, galectin-3 (Gal-3), galectin-9 (Gal-9), and galectin-10 (Gal-10) are the most extensively studied in human and animal asthma models. These galectins can affect T helper 2 (Th2) and non-Th2 inflammation, mucus production, airway responsiveness, and bronchial remodeling. Nevertheless, while higher Gal-3 and Gal-9 concentrations are associated with a stronger degree of Th-2 phlogosis, Gal-10, which forms Charcot–Leyden Crystals (CLCs), correlates with sputum eosinophilic count, interleukin-5 (IL-5) production, and immunoglobulin E (IgE) secretion. Finally, several galectins have shown potential in clinical response monitoring after inhaled corticosteroids (ICS) and biologic therapies, confirming their potential role as reliable biomarkers in patients with asthma.

## 1. Introduction

Asthma, a chronic respiratory disorder characterized by airway inflammation, increased airway responsiveness, and airway remodeling, poses a significant global health burden [1]. Despite recent advances in asthma research, the complex molecular mechanisms underlying the development and worsening of asthma remain only partially understood. From this perspective, the study of severe asthma endotypes paved the way for a more comprehensive approach towards asthma management, leading to the use of specific biomarkers in everyday clinical decisions. For instance, blood and sputum eosinophilic count, total serum immunoglobulin E (IgE), and fraction of exhaled nitric oxide (FeNO) have become irreplaceable tools when it comes to choosing treatments for severe asthma treatments and the monitoring of these treatments over time [2]. Moreover, after the introduction of monoclonal antibodies, some of these biomarkers have been proposed to assess the achievement of severe asthma clinical remission, which is now considered one of the main goals of biologic therapies [3,4,5].

Nevertheless, many molecular mechanisms related to specific asthma pathophysiologic traits, such as airway remodeling, bronchial hyperresponsiveness, and mucus production, are poorly understood. Serum and sputum periostin, Krebs von den Lungen-6 (KL-6), T-helper 2 (Th2) cytokines, and other biomarkers have been proposed to assess these features but their use in current clinical practice is still limited [6,7,8].

**Figure 1:**
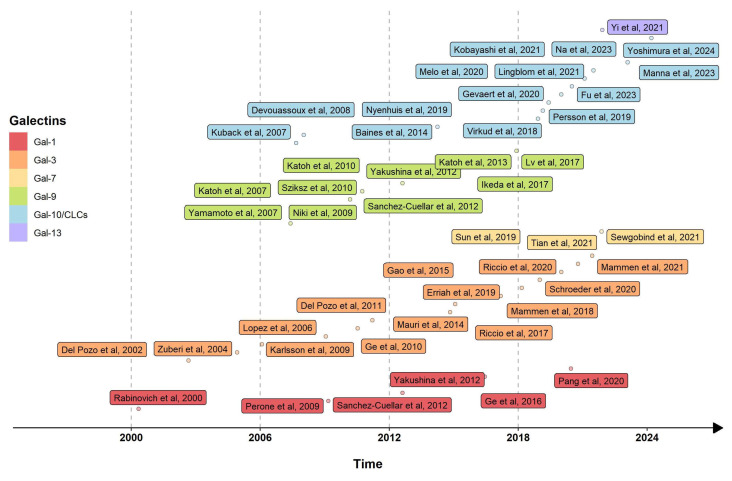
Chronological summary of the current literature on galectins and bronchial asthma [16,18,19,20,21,22,23,24,25,26,27,28,29,30,31,32,33,34,35,36,37,38,39,40,41,42,43,44,45,46,47,48,49,50,51,52,53,54,55,56,57,58,59,60,61,62,63]. Data were obtained from MEDLINE and EMBASE databases. No specific range of years was selected before the start of the literature revision.

In this context, a growing body of evidence has also implicated galectins, a family of evolutionarily conserved β-galactoside-binding proteins, in several processes of immune responses and inflammation development that could be related to asthma pathophysiology (see Figure 1) [9]. Galectins have emerged as crucial modulators of various cellular functions such as cell adhesion, migration, and apoptosis, as well as in many aspects of inflammatory response [10]. Consequently, several studies have demonstrated a possible role of galectins in several respiratory diseases like pulmonary fibrosis [11], COVID-19 disease [12,13,14], and lung cancer [15], highlighting their potential as biomarkers as well as possible treatable targets. Moreover, galectins could be involved in many crucial aspects of asthma development such as Th2 and non Th2 inflammation modulation, mucus production, and airway fibrotic remodeling [16,17]. Furthermore, some galectins could also be used to monitor the response to inhaled and biologic treatments, which would make this biomarker a useful tool for everyday clinical practice [18,19].

In this review, we described the relationship between galectins and asthma, exploring the impact of different galectin isoforms in the initiation, progression, and resolution of asthma-related inflammation.

## 2. Galectin-3

Galectin-3 (Gal-3) was initially identified as an IgE-binding protein [64]. It is widely distributed and can be found in extracellular spaces, cytoplasmic compartments, or nuclear regions. Like other galectin family members, Gal-3 lacks a secretion signal peptide that would guide its transport through the conventional endoplasmic reticulum-Golgi apparatus secretory pathway [65]. Through protein–protein interactions, Gal-3 has the capability to interact with numerous extracellular and/or intracellular proteins. Gal-3 is expressed in various tissues and cell types under normal conditions, including epithelial cells, dendritic cells, macrophages, and neutrophils. Acting as a chemoattractant and adhesion factor, Gal-3 plays a crucial role in the recruitment of monocytes and macrophages [16]. Gal-3 also plays a major role in various facets of asthma, including eosinophil recruitment, airway remodeling, and Th2 inflammation development [16].

In studies involving murine models, more emphasis has been placed on investigating the role of Gal-3 in the inflammatory response and its involvement in airway tissue remodeling in chronic allergic inflammation. Zuberi et al [20] analyzed the role of Gal-3 on airway inflammation in knockout Gal-3 gene (Gal-3 (−/−)) and wild-type mice (Gal-3 (+/+)). After ovalbumin (OVA) sensitization, both bronchoalveolar lavage (BAL) and histological analysis were performed on mouse lung samples fixed in formalin. As expected, BAL analysis of Gal-3 (−/−) mice showed a lower number of inflammatory cells, especially eosinophils and neutrophils [20]. However, the number of monocytes/macrophages did not significantly change between the two groups. Gal-3 (−/−) mice sensitized to OVA also exhibited significantly lower levels of goblet cell metaplasia and airway responsiveness compared to Gal-3 (+/+) mice [20]. Following the path linking Gal-3 to eosinophilic inflammation, Ge and colleagues reported lower levels of interleukin-5 (IL-5) and interleukin-13 (IL-13) in Gal-3 (−/−) mice, which are pivotal cytokines for Th2 inflammation development. Furthermore, after the OVA challenge, Gal-3 knockout mice showed a reduction in lung eotaxin-1 levels, which is responsible for the activation and recruitment of eosinophils in the allergic response. Such a result may be explained due to a reduced infiltration of lymphocytes, alveolar macrophages and eosinophils into the airways, which are known to release eotaxin-1. Alternatively, this result could be secondary to a decreased production of this chemokine by lung epithelial cells induced by the lower levels of IL-13 [21].

Gal-3 has also been shown to be involved in mucus production and airway remodeling. Ge et al [21] highlighted a lower count of periodic acid-Schiff (PAS) positive mucus-producing cells in allergen-challenged Gal-3 (−/−) mice compared to wild-type mice. Moreover, Gal-3 genes suppression resulted in a less intense peribronchial fibrosis [21], probably due to a more pronounced Th1 immune response and a less severe Th2 inflammation [65]. From this perspective, Gal-3 has several important roles in non-Th2 inflammation and mucus production. As reported by Mammen and colleagues [22], knockout mice for tissue inhibitor of metalloproteinase 1 (TIMP-1) tend to have greater blood and lung Gal-3 concentrations compared to wild-type mice. After OVA sensitization and TIMP-1 inhibition, the increase in Gal-3 concentrations leads to the lung overexpression of interleukin-17 (IL-17) genes, with a reduced transcription of the mucin family genes *MUC5AC* and *MUC5B* [23]. This IL-17/Gal-3 interaction could be promoted by the increased transforming growth factor β1 (TGF-β1) gene expression induced by TIMP-1 inhibition and OVA sensitization [22]. Moreover, Gal-3 inhibition significantly increases nuclear factor kappa-light-chain-enhancer of activated B cells (NF-κβ) gene expression, which has a crucial role in allergic inflammation development and mucin production via IL-17A [66,67]. IL-17 is a pivotal cytokine involved in allergic response, non-Th2 inflammation, mucus production and airway remodeling in asthma [23,68]. From this perspective, these results could explain the complex interactions between Gal-3, IL-17, TGF-β1 and NF-κβ in asthma pathophysiology.

Another important aspect of Gal-3 functions deals with its role as the mediator of the lung epithelial cells–immune system axis. Gal-3 administration can activate interleukin-6 (IL-6) and tumor necrosis factor-α (TNF-α) production in dendritic cells with an IgE-independent mechanism, as well as basophils proliferation via an IgE-dependent bond [24]. Gal-3 can also take part in neutrophils activation and recruitment in the presence of bacterial or fungal infections [69], which can be crucial triggers for asthma exacerbations [2]. In rodent models, the presence of *Streptococcus pneumoniae* in alveolar spaces induces Gal-3 release from macrophages, with a consequent neutrophils’ extravasation mediated by their adhesion to Gal-3 [70]. Similarly, after *Aspergillus Fumigatus* exposition, Gal-3 expression is markedly increased in serum and on immune cells, without showing a direct fungicidal function but inducing neutrophils activation and extravasation [71]. Gal-3 can also affect macrophages efferocytosis, which can result in the accumulation of apoptotic cells in the lung tissue and, consequently, could lead to the release of cytokines, reactive oxygen species, and other inflammatory mediators. Patients with non-eosinophilic asthma show the greatest rate of efferocytosis deficiency but also a major improvement in this cellular mechanism after the administration of exogenous Gal-3 [25].

Several interesting findings on Gal-3 biomechanisms also arise from studies on Gal-3 plasmid gene therapy. As described by Del Pozo and colleagues [26] in murine models, Gal-3 gene therapy improves acute airway obstruction and bronchial inflammation through the downregulation of IL-5, with a consequent drop in BAL T lymphocytes and eosinophils. Interestingly, the use of Gal-3 therapy has also shown positive results on murine models simulating chronic asthma, with a significant reduction in key BAL cytokines like IL-5, interleukin-4 (IL-4), and interleukin-10 (IL-10). Additionally, intrapulmonary gene therapy influences allergen-induced eosinophilia in both BAL and peripheral blood, as demonstrated by the decreased eosinophils concentration in these samples. From a histological standpoint, these results were confirmed by the reduction in PAS-positive cells (typical of mucus cell metaplasia) and by the absence of peribronchial and perivascular inflammatory infiltrates in mice treated with Gal-3 [27]. However, these findings might not represent the effect of endogenous Gal-3, as its role in Th1/Th2 immune and inflammatory responses may vary according to the experimental model used to study allergic asthma and because the effects of exogenously administered substances can differ from those of endogenous ones [27]. Currently, there are no commercially available therapeutic agents or inhibitors specifically targeting endogenous Gal-3. The development of selective inhibitors is challenging due to the weak protein–carbohydrate interactions and the extensive sequence similarity in the carbohydrate recognition domain (CRD) of galectins. Nevertheless, there have been advancements in the development of selective Gal-3 antagonists or inhibitors effective in mitigating lung fibrosis, currently undergoing preclinical or phase I testing [72].

Another interesting aspect of the immune-modulation led by Gal-3 is related to its relationship with suppressor of cytokine signaling (SOCS), a family of proteins which regulates cytokine action and immune crosstalk of complex signal network mechanisms, thereby influencing the Th1/Th2 balance [73]. Mice treated with Gal-3 gene therapy exhibited a marked downregulation of SOCS1 and SOCS3, resulting in lower levels of IL-10 and TGF-β [74]. These findings imply that downregulation of SOCS1 and 3 by Gal-3 treatment might represent a promising therapeutic strategy for allergic diseases.

As seen in several murine models, Gal-3 can influence the Th1/Th2 balance, as it is directly connected to allergic and eosinophilic inflammation [28]. However, it is well known that approximately 50% of asthma cases are not Th2-mediated. Gao and colleagues [28] highlighted how patients with neutrophilic asthma, defined by neutrophils ≥61% in sputum samples, had lower levels of Gal-3 than patients with paucigranulocytic (sputum eosinophils ≤ 3% and neutrophils < 61%) or eosinophilic (sputum eosinophils ≥3%) asthma. Moreover, patients with neutrophilic asthma also showed increased levels of Gal-3 binding protein (Gal-3BP), which can decrease Gal-3 activity and Th2 inflammation [28]. Considering the role of Gal-3 in enhancing macrophage-mediated apoptotic neutrophils clearance [29], it has been hypothesized that Gal-3/Gal-3BP could lead to an impaired ability of leukocytes to remove apoptotic neutrophils, thereby promoting inflammation persistence [28].

Gal-3 has also been addressed as a robust biomarker for severe asthma biologic treatment response. This protein has been identified as a valuable predictor of clinical and functional improvement in patients receiving up to 3 years of anti-IgE therapy [18]. Proteomic analysis has revealed that baseline Gal-3 levels in bronchial samples are indicative of a positive response to omalizumab on airway remodeling, probably due to the interaction of Gal-3 with the complex high-affinity cell-surface receptor for the Fc region of antigen-specific IgE molecules (IgE–FcεR1), which is believed to cause its dissociation and a better response to anti-IgE treatment [30]. Similarly, in the PROXIMA study, a multicenter, single-arm, observational study conducted in 25 Italian centers, patients with baseline serum Gal-3 concentrations ≥ 11 ng/mL had a higher likelihood of being a super responder or a functional responder after a 12-month course of anti-IgE treatment [31].

In conclusion, Gal-3 is involved in many pivotal aspects of asthma pathophysiology such as allergic response, eosinophils activation, and non-Th2 inflammation. For this reason, Gal-3 can be considered a promising biomarker for asthma diagnosis and monitoring over time.

## 3. Galectin-9

Galectin-9 (Gal-9) is expressed in different cells associated with the immune system, having a role in both innate and adaptive immunity by regulating cell proliferation, differentiation, cellular signaling, RNA splicing, apoptosis, and cellular motility [72]. Gal-9 express its role in physiologic and pathologic conditions, as demonstrated by its higher blood concentrations in acute and chronic infectious disease [75]. Gal-9 has a role also in macrophage polarization (M1-type and M2-type) in several stages of an inflammatory response, with increased levels of Gal-9 inducing TGF-β, IL-10, and signal transducer and activator of transcription 3 (STAT3) production after M2-type macrophages differentiation. Moreover, lower levels of Gal-9 in macrophages are associated with a marked expression of TNF-α, IL-6, STAT1, and NF-κβ genes, which are directly related to M1-type macrophages activity [32].

The role of Gal-9 in viral respiratory infection and asthma exacerbation has been investigated in a study evaluating the possible preventive effect of this protein on airway hyperresponsiveness in a murine model of antigen-induced asthma. Katoh et al analyzed the inflammation of BAL of mice with antigen-induced chronic asthma challenged with the administration of synthetic dsRNA and polyinosinic-polycytidylic acid (PolyIC) to replicate the effect of a virus infection. The results showed that Gal-9 administration leads to a significant reduction in airway inflammation obtained by the suppression of PolyIC and the production of specific cytokine cluster including TNF-α, regulated upon activation, normal T cell expressed and presumably secreted (RANTES) and interferon gamma-induced protein 10 (IP-10) [33]. On the other hand, chronic models of asthma developed on guinea pigs revealed that Gal-9 has a chemoattractant function for eosinophils, influencing eosinophil peroxidase (EPO) release and T lymphocytes apoptosis in the sensitized mice group [34]. Interestingly, in murine models of allergic asthma, Gal-9 showed a pivotal role in Th2 inflammation with several molecular mechanisms. In fact, Gal-9 can bind CD44, a glycosylated adhesion molecule highly expressed on many human cells and involved in lymphocytes migration and airway hyperresponsiveness (AHR) development in asthma [35,76]. Gal-9 can inhibit CD44 interaction with hyaluronan (HA), decreasing lymphocytes and eosinophils airway migration. As a direct consequence, Gal-9 can also reduce AHR by modulating the CD44-dependent leukocyte recognition of the extracellular matrix. Furthermore, mice treated with exogenous Gal-9 reduces their lung concentrations of IL-5, IL-13 and eotaxin, which are key elements for eosinophils migration and activation [35]. Similarly, Gal-9 administration in a guinea pigs asthma model had a suppressing effect on eosinophils accumulation in lungs, due to the suppression of their CD44-dependent migration.

Another interesting aspect regards the relationship between Gal-9 concentrations, mast cells degranulation, and IgE activity. In guinea pigs challenged with a 2% OVA, the increase in airway resistances and BAL eosinophils’ concentrations were mitigated by Gal-9 administration [36]. Moreover, in rat basophilic leukemia RBL-2H3 cells, Gal-9 substantially reduced β-hexosaminidase (β-HEX), histamine, and leukotriene C4 secretion. Finally, Gal-9 revealed a strong affinity for IgE, being able to inhibit IgE-antigen complex formation and further reducing mast-cell degranulation [36].

The role of exogenous administration of Gal-9 was also studied in a murine model reproducing allergic immunity after 5 weeks of Dermatophagoides farinae sensitization. After the administration of an allergen-specific sublingual immunotherapy, mice receiving adjuvant Gal-9 showed an improved airway hyperresponsiveness along with a significant reduction in IL-5, IL-13, and IgE BAL and blood concentrations, demonstrating the potential role of Gal-9 as a treatable trait for both allergic and eosinophilic asthma inflammation [37]. Nevertheless, while exogenous administration of Gal-9 could reduce the recruitment and activation of eosinophils granulocytes and, consequentially, airway inflammation, in physiological concentrations, this protein seems to induce the chemotaxis of the same cells, promoting eosinophilic inflammation [34,38]. A possible explanation for this phenomenon could be related to a dose-dependent mechanism, with Gal-9 physiologic levels increasing eosinophilic inflammation while higher concentrations of Gal-9 suppressing their activity and chemotaxis [38]. Sziksz et al [38] also investigated the role of Gal-9 in airway hyperresponsiveness on a group of mice sensitized with OVA, addressing the increased levels of lung Gal-9 as a potential factor influencing asthma progression. It has been hypothesized that Gal-9 could lead to a Th1/Th2 disbalance, with a dominance of Th2 cytokines and a pronounced Th1 apoptosis [77]. This evidence seems to support the possible use of exogenous Gal-9 to limit the T-cell activity and improve asthma control, since Gal-9 can effectively reduce IgE-mediated mast cells degranulation, eosinophils chemotaxis and activation and allergic response.

Finally, the role of Gal-9 has also been studied in patients with asthma. While cells from sputum samples did not reveal different levels of Gal-9 mRNA expression in asthmatic patients, Gal-9 surface expression on leucocytes was reduced in the presence of asthma, independently from atopic inflammation [39]. Interestingly, Gal-9 could also induce IL-10 production from mononuclear cells, confirming the role of this protein in non-Th2 inflammation development as well [39]. These effects on Th2 and non-Th2 inflammation, along with the impact on AHR, could make Gal-9 a suitable biomarker for asthma management, as well as a possible target for future personalized therapeutic approaches [40,78].

## 4. Galectin-10

Galectin-10 (Gal-10) is highly expressed in human eosinophils but has also been identified in basophils, macrophages, and T lymphocytes [41,79]. Differently from other proteins, Gal-10 is not stored in eosinophils’ primary granules nor secreted via classic degranulation processes [42,80], but it has higher cytosolic concentrations, being also present in eosinophils’ nucleus and cellular membrane fragments [81,82]. These findings would suggest many possible roles of Gal-10 in nuclear translation/transcription processes, cytoplasmic transport, and cell-to-cell interaction. Human Gal-10 contains a not-glycosylated CRD, which allows for its crystallization when reaching specific concentrations [79]. In fact, Gal-10 can spontaneously form the Charcot–Leyden crystals (CLCs), hexagonal and bipyramidal colorless structures first described in 1853 by Jean-Martin Charcot in spleen and heart samples of leukemia patients [17]. Later, in 1996, Dyer et al demonstrated that the primary structure of CLCs highly resemble Gal-1 and Gal-3 [83], allowing for the classification of CLCs among the galectin family. However, the extracellular crystallization of Gal-10 is still not completely understood due to the lack of an equivalent rodent model for this protein. Nevertheless, the administration of CLCs in mice can induce monocyte/macrophages, dendritic cells, and neutrophils proliferation via TNF-α, IL-1β, and IL-6 production [43]. Interestingly, while molecules like lactose or galactose can induce the development of CLCs through an electrostatic potential shift during Gal-10 dimerization [44], Glutathione appears to inhibit the formation of CLCs in animal models, binding the CRD of CLCs and preventing Gal-10 crystallization [45]. However, apart from its release after crystallization, Gal-10 can also form eosinophil extracellular traps (EETs) along with histone-rich DNA and other cytoplasmic proteins from secondary granules, being secreted by active eosinophils or after their cytolysis (ETosis) [84,85]. As reported by Yoshimura and colleagues [46], Gal-10 is hyperrepressed in chronic rhinosinusitis with nasal polyposis (CRwNP) extracellular vesicles (EVs) and can participate in the ETosis process. Moreover, ETosis also characterizes eosinophilic granulomatosis with polyangiitis (EGPA), where Gal-10 EETs can cause small vessels damage and platelets aggregation [86]. Moreover, after thymic stromal lymphopoietin (TSLP) and/or IL-5 stimulation, Gal-10 released with EETs can induce synapses development between eosinophils and CD4+/CD8+ lymphocytes, probably promoting their suppression [47].

CLCs have been identified in several diseases linked to eosinophilic inflammation such as celiac disease [87], hypereosinophilic syndrome [88] and Aspergillus diseases [89]. CLCs are also highly expressed in EGPA and closely related to disease severity, vascular damage and IL-5 serum concentrations [90]. Gal-10 also appears to have a role in the development of seasonal allergic rhinitis in pediatric patients, especially considering its relationship with IL-5 production and Th2 inflammation [48]. Moreover, the gene expression of CLCs is closely related to total IgE serum concentrations, which are also a powerful trigger for eosinophils activation binding FcεRII receptor [49].

Compared to Gal-3 and Gal-9, which can suppress Th-2 inflammation at higher concentrations, Gal-10 shows an opposite behavior, being strongly associated with blood and sputum eosinophilia [50], IL-5 secretion [49,90], and IgE production [49]. Despite the apparent contradiction, a possible explanation could be found in the relationship between CLCs and macrophages activation. Gal-10 can induce IL-1β production and secretion after the phagocytosis of CLCs by macrophages, which can promote and sustain Th2 inflammation [51]. IL-1β release seems to be secondary to NOD-, LRR-, and pyrin domain-containing protein 3 (NLRP3) inflammasome activation, which can be promoted in vivo by CLCs without a priming process [51].

Considering how Gal-10 modulates Th2 inflammation, several studies have investigated the possible role of this biomarker in bronchial asthma. Baines and colleagues [19], after the analysis of 6 signature sputum genes, reported an increased CLC gene expression in patients with eosinophilic asthma, while inhaled fluticasone administration led to a significant reduction in Gal-10 production. In this study, the use of azithromycin was associated with a reduction in asthma exacerbation rate without affecting the predictive ability of the studied genes, underscoring the possible clinical utility of sputum analysis of these biomarkers [19]. Interestingly, this 6-genes signature model has also shown good reliability discriminating eosinophilic, non-eosinophilic and paucigranulocytic asthma endotypes as well as clinically significant improvements in forced expiratory volume in 1 second (FEV1), asthma control questionnaire (ACQ) and FeNO values after inhaled fluticasone administration [19].

A significant response to treatment has also been described after anti-IL5 monoclonal antibody administration since serum Gal-10 significantly and rapidly decreases 4 weeks after the initial injection of mepolizumab 100 mg [52]. Moreover, other independent cohorts reported the overexpression of Gal-10 mRNA in patients with aspirin-induced asthma [53,91], suggesting a potential role of this protein in aspirin-induced respiratory diseases.

The clinical role of CLCs’ evaluation has also been studied considering the presence of Gal-10 in extracellular vesicles (EVs). In patients with eosinophilic asthma, EVs containing Gal-10 revealed a higher diagnostic correlation with blood eosinophilic count compared to EPO EVs concentrations [46]. Furthermore, Gal-10 EVs levels had the strongest correlation with mucus score, FEV1 and bronchial wall thickening, with a consistent expression of Gal-10 not only in EVs but also in bronchial tissue [46].

Finally, Gal-10 can also play a major role in mucus production. In patients with Th2 asthma, CLCs correlate with sputum eosinophils [50], which are known to be a crucial element for mucus plug development [92]. Furthermore, in rodent model reproducing allergic asthma, exogenous administration of crystallized Gal-10 could induce *MUC5A* gene expression, promoting mucus formation and mucus plugs deposition in small airways [93]. CLCs may also modify mucus rheologic features through their CRD, which can provide more structure and tenacity to respiratory secretions, increasing mucus plugs development [94]. This aspect has also been studied in the context of a Th1 inflammation where, in murine models, CLCs can induce TNF-α, IL-1β and IL-6 production, causing neutrophils, macrophages and dendritic cells proliferation [43]. Similarly, in patients with CRwNP, recombinant CLCs’ administration induces serum IL-1, IL-6 and TNF-α release and neutrophilic extracellular traps (NETs) development [54], which can increase mucus viscoelasticity and cause airway mucus obstruction [95]. Therefore, the abundance of CLCs in mucus plugs could empower both Th1 and Th2 inflammation, resulting in even more mucus production and feeding a vicious circle which can worsen asthma and CRwNP management [93].

## 5. Other Galectins

While Gal-3, Gal-9, and Gal-10 have been more extensively studied, there are other isoforms, like galectin-1 (Gal-1), galectin-7 (Gal-7), and galectin-13 (Gal-13), with less available data in scientific literature. For this reason, we gathered the main findings regarding these galectins in this specific section.

### 5.1. Galectin-1

Gal-1 plays a pivotal role in different phases of the inflammatory response, having immunosuppressive effects after its administration in animal models simulating inflammatory diseases [55,56]. Gal-1 also participate in the shift from a CD4+ T cell response towards the Th2 and Treg cells subsets. Yakushina et al investigated in vitro the effects of Gal-1 on mRNA expression levels of T-box transcription factor (TBX21), GATA-binding protein 3 (GATA3), forkhead box P3 (FOXP3) and RAR-related orphan receptor C (RORC), which drive CD4+ T cell differentiation into Th1, Th2, Treg and Th17 lymphocytes [57]. Interestingly, Gal-1 reduces TBX21 and RORC expression, increasing GATA-3-FOXP3 transcription and tilting the balance of Th1 lymphocytes differentiation to Th2 and Treg cells. These findings confirmed Gal-1 involvement in Th2 inflammation development, highlighting its possible role in asthma pathophysiology [57]. In fact, patients with asthma have lower sputum mRNA levels for Gal-1 and Gal-3 compared to healthy subjects (*p* = 0.08 and *p* < 0.05, respectively), as well as a lower expression of Gal-1 and Gal-9 on [39]. Differently, Gal-3 was also expressed on neutrophils’ membrane independently from the presence of an atopic trait. Interestingly, after the monocytes stimulation with lipopolysaccharide (LPS), the presence of Gal-1 induces a sustained production of IL-10, which is a known cytokine for the downregulation of Th2 inflammatory response [39].

Following these findings, the role of Gal-1 in allergic airway inflammation has been further explored in a study on a mouse model of allergic asthma [58]. Exposure to OVA led to an increased Gal-1 expression on airway epithelial cells, smooth muscle cells, endothelial cells, inflammatory cells and in the extracellular space compared to the control population. Additionally, the expression of cell-surface glycans on murine eosinophils allowed for Gal-1 binding, causing the reduction in eosinophils chemotaxis and, at concentrations ≥1.0 μM, inducing eosinophils apoptosis [58]. Finally, Gal-1 can also inhibit platelet-derived growth factor BB (PDGF-BB) and, consequentially, the proliferation and migration of airway smooth muscle cell. Data from in vitro analysis suggest that this effect could probably rely on the inactivation of the phosphatidylinositol 3-kinase(PI3K)-Akt (PI3K/Akt) signaling pathway, which is obtained at high concentrations of Gal-1 [59]. Considering these findings, Gal-1 production seems to exhibit a dual role in patients with asthma, promoting lung inflammation but also decreasing airway smooth cells activity. However, eosinophils depletion after Gal-1 binding to eosinophilic membrane glycans in sensitized mice [58] as well as IL-10 production after LPS exposure [39] would suggest a specific role for Gal-1 in the orchestration of Th2 inflammation in patients with asthma, which should be further explored in future studies.

### 5.2. Galectin-7

Gal-7 is involved in apoptosis and in cells proliferation, differentiation, adhesion and migration. In mice, the overexpression of Gal-7 was linked to the development of abnormal airway structures (thin an altered epithelium) in embryos and after birth, suggesting that this protein is involved in the architectural airway defects related to asthma development [60]. The effects of Gal-7 on airway epithelial barrier were also described on transgenic murine models, where Gal-7 overexpression led to a reduction in pseudostratified columnar ciliated epithelium and to an increase in monolayer cells with irregular intercellular spaces aspects [61]. Moreover, cell junctions were altered as well, being more susceptible to critical damage when exposed to respiratory syncytial virus (RSV) or OVA [61]. Finally, Gal-7 inhibition is proven to reduce TGF-β1-induced apoptosis of human bronchial epithelial cell through the inhibition of the Jun N-terminal kinase (JNK) pathway [62].

### 5.3. Galectin-13

Gal-13 is a member of the lectin family that does not exist in non-human species, with an important research limitation due to the lack of a reliable animal model. Nevertheless, it is known that Gal-13 contributes to asthma inflammation, as proven by a study conducted on 54 subjects with asthma showing significantly higher level of Gal-13 transcript in bronchial brushing [63]. Moreover, immunohistochemical staining on airway biopsy revealed that Gal-13 is mainly localized in epithelial cells and submucosal gland cells in asthmatic patients, which would suggest its potential role in epithelial barrier signaling and mucus production [63].

Levels of epithelial Gal-13 were also confronted with the sputum and blood eosinophilic count, with the number of eosinophils in bronchial submucosa and with FeNO levels, with a sensitive correlation between Gal-13 concentrations and eosinophils activity [63]. Exploring the mechanism by which Gal-13 participates in airway eosinophilic inflammation, Lingling et al. found that a possible answer is the promoting action of this protein on the expression of monocyte chemotactic protein (MCP-1) and eotaxin-1 in airway epithelial cells. Moreover, preliminary data suggest that Gal-13 serum levels could predict bronchial responses to inhaled corticosteroids therapy [63]. Future studies could better address the role and the potential of Gal-13 in the diagnosis and follow-up for bronchial asthma.

## 6. Summary

**Figure 2:**
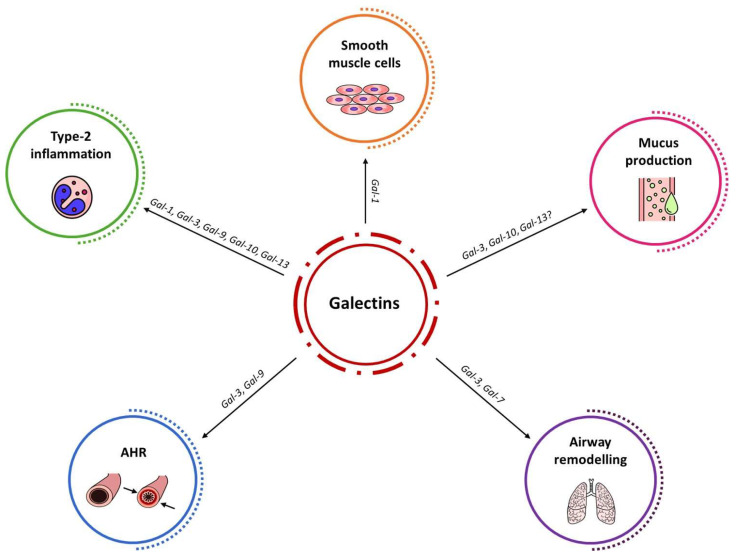
Galectins’ role in main aspects of asthma pathophysiology. AHR: Airway hyperresponsiveness.

Galectins represents a heterogeneous class of proteins with pleiotropic functions on many aspects of immune response, cellular proliferation, and genes expression. In patients with asthma, many galectins promote Th2 inflammation and eosinophils activation, with several potential repercussions on airway hyperresponsiveness, mucus production, and bronchial remodeling (See Figure 2). Furthermore, some of these glycan-binding proteins can activate Th1 response in specific contexts, generating many complex interactions in innate and adaptive immune response.

Despite the current evidence, many aspects of galectins’ roles are still poorly understood. A considerable part of our knowledge regarding their function has been obtained from animal models, limiting the interpretability of the available information. Moreover, for Gal-10 and Gal-13, the absence of their endogenous expression in animal models further limits our research potential, leaving fewer opportunities for a deeper understanding of which pathophysiological mechanisms are sustained by these proteins. Finally, many facets of the use of galectins in everyday clinical practice are still to be explored, as there is very little evidence on their possible use as biomarkers in asthma diagnosis and long-term management. This aspect could be of major interest in future research, especially considering the recent advances in asthma treatment with monoclonal antibodies. To date, there are only a few studies describing the predictive power of galectins after ICS or biologics (omalizumab, mepolizumab) treatment [19,52], so many aspects concerning their relationship with ICS escalation/de-escalation [96] and biologics administration/switch [97] remain poorly understood. Moreover, considering the role of some galectins (Gal-3, Gal-9, Gal-10) on both Th1 and Th2 inflammation, it would be interesting to investigate whether these proteins could reliably be used in the presence of asthma and Th1-driven comorbidities such as respiratory infections or bronchiectasis [98].

## 7. Conclusions

Galectins appear to be involved in many aspects of asthma development, representing a valuable biomarker for the assessment of disease pathophysiology and treatment success. Future prospective studies should specifically evaluate the relationship between galectins and main asthma clinical and functional outcomes, also focusing on galectins’ impact on those pivotal asthma features which have been less extensively characterized, such as airway responsiveness and remodeling.

## References

[1] Reports—Global Initiative for Asthma—GINA. https://ginasthma.org/reports/.

[2] Chung K.F., Wenzel S.E., Brozek J.L., Bush A., Castro M., Sterk P.J., Adcock I.M., Bateman E.D., Bel E.H., Bleecker E.R. (2013). International ERS/ATS guidelines on definition, evaluation and treatment of severe asthma. Eur. Respir. J..

[3] Menzies-Gow A., Bafadhel M., Busse W.W., Casale T.B., Kocks J.W., Pavord I.D., Szefler S.J., Woodruff P.G., de Giorgio-Miller A., Trudo F. (2020). An expert consensus framework for asthma remission as a treatment goal. J. Allergy Clin. Immunol..

[4] Portacci A., Dragonieri S., Carpagnano G.E. (2023). Super-Responders to Biologic Treatment in Type 2–High Severe Asthma: Passing Fad or a Meaningful Phenotype?. J. Allergy Clin. Immunol. Pr..

[5] Carpagnano G.E., Portacci A., Nolasco S., Detoraki A., Vatrella A., Calabrese C., Pelaia C., Montagnolo F., Scioscia G., Valenti G. (2024). Features of severe asthma response to anti-IL5/IL5r therapies: Identikit of clinical remission. Front. Immunol..

[6] Vianello A., Guarnieri G., Achille A., Lionello F., Lococo S., Zaninotto M., Caminati M., Senna G. (2023). Serum biomarkers of remodeling in severe asthma with fixed airway obstruction and the potential role of KL-6. Clin. Chem. Lab. Med. (Cclm).

[7] Gordon E.D., Sidhu S.S., Wang Z., Woodruff P.G., Yuan S., Solon M.C., Conway S.J., Huang X., Locksley R.M., Fahy J.V. (2011). A protective role for periostin and TGF-β in IgE-mediated allergy and airway hyperresponsiveness. Clin. Exp. Allergy.

[8] Khalfaoui L., Symon F.A., Couillard S., Hargadon B., Chaudhuri R., Bicknell S., Mansur A.H., Shrimanker R., Hinks T.S.C., Pavord I.D. (2022). Airway remodelling rather than cellular infiltration characterizes both type2 cytokine biomarker-high and -low severe asthma. Allergy.

[9] Cooper D.N. (2002). Galectinomics: Finding themes in complexity. Biochim. et Biophys. Acta (BBA) Gen. Subj..

[10] Thiemann S., Baum L.G. (2016). Galectins and Immune Responses—Just How Do They Do Those Things They Do?. Annu. Rev. Immunol..

[11] Hirani N., MacKinnon A.C., Nicol L., Ford P., Schambye H., Pedersen A., Nilsson U.J., Leffler H., Sethi T., Tantawi S. (2020). Target inhibition of galectin-3 by inhaled TD139 in patients with idiopathic pulmonary fibrosis. Eur. Respir. J..

[12] Portacci A., Diaferia F., Santomasi C., Dragonieri S., Boniello E., Di Serio F., Carpagnano G.E. (2021). Galectin-3 as prognostic biomarker in patients with COVID-19 acute respiratory failure. Respir. Med..

[13] Gaughan E.E., Quinn T.M., Mills A., Bruce A.M., Antonelli J., MacKinnon A.C., Aslanis V., Li F., O’connor R., Boz C. (2023). An Inhaled Galectin-3 Inhibitor in COVID-19 Pneumonitis: A Phase Ib/IIa Randomized Controlled Clinical Trial (DEFINE). Am. J. Respir. Crit. Care Med..

[14] Portacci A., Amendolara M., Quaranta V.N., Iorillo I., Buonamico E., Diaferia F., Quaranta S., Locorotondo C., Schirinzi A., Boniello E. (2024). Can Galectin-3 be a reliable predictive biomarker for post-COVID syndrome development?. Respir. Med..

[15] Yang R., Sun L., Li C.-F., Wang Y.-H., Yao J., Li H., Yan M., Chang W.-C., Hsu J.-M., Cha J.-H. (2021). Galectin-9 interacts with PD-1 and TIM-3 to regulate T cell death and is a target for cancer immunotherapy. Nat. Commun..

[16] Gao P., Simpson J.L., Zhang J., Gibson P.G. (2013). Galectin-3: Its role in asthma and potential as an anti-inflammatory target. Respir. Res..

[17] Tomizawa H., Yamada Y., Arima M., Miyabe Y., Fukuchi M., Hikichi H., Melo R.C.N., Yamada T., Ueki S. (2022). Galectin-10 as a Potential Biomarker for Eosinophilic Diseases. Biomolecules.

[18] Riccio A.M., Mauri P., De Ferrari L., Rossi R., Di Silvestre D., Benazzi L., Chiappori A., Negro R.W.D., Micheletto C., Canonica G.W. (2017). Galectin-3: An early predictive biomarker of modulation of airway remodeling in patients with severe asthma treated with omalizumab for 36 months. Clin. Transl. Allergy.

[19] Baines K.J., Simpson J.L., Wood L.G., Scott R.J., Fibbens N.L., Powell H., Cowan D.C., Taylor D.R., Cowan J.O., Gibson P.G. (2014). Sputum gene expression signature of 6 biomarkers discriminates asthma inflammatory phenotypes. J. Allergy Clin. Immunol..

[20] Zuberi R.I., Hsu D.K., Kalayci O., Chen H.-Y., Sheldon H.K., Yu L., Apgar J.R., Kawakami T., Lilly C.M., Liu F.-T. (2004). Critical Role for Galectin-3 in Airway Inflammation and Bronchial Hyperresponsiveness in a Murine Model of Asthma. Am. J. Pathol..

[21] Ge N.X., Bahaie N.S., Na Kang B., Hosseinkhani M.R., Gil Ha S., Frenzel E.M., Liu F.-T., Rao S.P., Sriramarao P. (2010). Allergen-Induced Airway Remodeling Is Impaired in Galectin-3–Deficient Mice. J. Immunol..

[22] Mammen M.J., Sands M.F., Abou-Jaoude E., Aalinkeel R., Reynolds J.L., Parikh N.U., Sharma U., Schwartz S.A., Mahajan S.D. (2017). Role of Galectin-3 in the pathophysiology underlying allergic lung inflammation in a tissue inhibitor of metalloproteinases 1 knockout model of murine asthma. Immunology.

[23] Mammen M.J., Ali J., Aurora A., Sharma U.C., Aalinkeel R., Mahajan S.D., Sands M., Schwartz S.A. (2021). IL-17 Is a Key Regulator of Mucin-Galectin-3 Interactions in Asthma. Int. J. Cell Biol..

[24] Schroeder J.T., Adeosun A.A., Bieneman A.P. (2020). Epithelial Cell-Associated Galectin-3 Activates Human Dendritic Cell Subtypes for Pro-Inflammatory Cytokines. Front. Immunol..

[25] Erriah M., Pabreja K., Fricker M., Baines K.J., Donnelly L.E., Bylund J., Karlsson A., Simpson J.L. (2019). Galectin-3 enhances monocyte-derived macrophage efferocytosis of apoptotic granulocytes in asthma. Respir. Res..

[26] del Pozo V., Rojo M., Rubio M.L., Cortegano I., Cárdaba B., Gallardo S., Ortega M., Civantos E., López E., Martín-Mosquero C. (2002). Gene Therapy with Galectin-3 Inhibits Bronchial Obstruction and Inflammation in Antigen-challenged Rats through Interleukin-5 Gene Downregulation. Am. J. Respir. Crit. Care Med..

[27] López E., del Pozo V., Miguel T., Sastre B., Seoane C., Civantos E., Llanes E., Baeza M.L., Palomino P., Cárdaba B. (2006). Inhibition of Chronic Airway Inflammation and Remodeling by Galectin-3 Gene Therapy in a Murine Model. J. Immunol..

[28] Gao P., Gibson P.G., Baines K.J., A Yang I., Upham J.W., Reynolds P.N., Hodge S., James A.L., Jenkins C., Peters M.J. (2015). Anti-inflammatory deficiencies in neutrophilic asthma: Reduced galectin-3 and IL-1RA/IL-1β. Respir. Res..

[29] Karlsson A., Christenson K., Matlak M., Björstad Å., Brown K.L., Telemo E., Salomonsson E., Leffler H., Bylund J. (2008). Galectin-3 functions as an opsonin and enhances the macrophage clearance of apoptotic neutrophils. Glycobiology.

[30] Mauri P., Riccio A.M., Rossi R., Di Silvestre D., Benazzi L., De Ferrari L., Negro R.W.D., Holgate S.T., Canonica G.W. (2014). Proteomics of bronchial biopsies: Galectin-3 as a predictive biomarker of airway remodelling modulation in omalizumab-treated severe asthma patients. Immunol. Lett..

[31] Riccio A.M., Mauri P., De Ferrari L., Rossi R., Di Silvestre D., Bartezaghi M., Saccheri F., Canonica G.W. (2020). Plasma Galectin-3 and urine proteomics predict FEV1 improvement in omalizumab-treated patients with severe allergic asthma: Results from the PROXIMA sub-study. World Allergy Organ. J..

[32] Lv R., Bao Q., Li Y. (2017). Regulation of M1-type and M2-type macrophage polarization in RAW264.7 cells by Galectin-9. Mol. Med. Rep..

[33] Katoh S., Shimizu H., Obase Y., Oomizu S., Niki T., Ikeda M., Mouri K., Kobashi Y., Hirashima M., Oka M. (2013). Preventive effect of galectin-9 on double-stranded RNA-induced airway hyperresponsiveness in an exacerbation model of mite antigen-induced asthma in mice. Exp. Lung Res..

[34] Yamamoto H., Kashio Y., Shoji H., Shinonaga R., Yoshimura T., Nishi N., Nabe T., Nakamura T., Kohno S., Hirashima M. (2007). Involvement of Galectin-9 in Guinea Pig Allergic Airway Inflammation. Int. Arch. Allergy Immunol..

[35] Katoh S., Ishii N., Nobumoto A., Takeshita K., Dai S.-Y., Shinonaga R., Niki T., Nishi N., Tominaga A., Yamauchi A. (2007). Galectin-9 Inhibits CD44–Hyaluronan Interaction and Suppresses a Murine Model of Allergic Asthma. Am. J. Respir. Crit. Care Med..

[36] Niki T., Tsutsui S., Hirose S., Aradono S., Sugimoto Y., Takeshita K., Nishi N., Hirashima M. (2009). Galectin-9 Is a High Affinity IgE-binding Lectin with Anti-allergic Effect by Blocking IgE-Antigen Complex Formation. J. Biol. Chem..

[37] Ikeda M., Katoh S., Shimizu H., Hasegawa A., Ohashi-Doi K., Oka M. (2017). Beneficial effects of Galectin-9 on allergen-specific sublingual immunotherapy in a Dermatophagoides farinae -induced mouse model of chronic asthma. Allergol. Int..

[38] Sziksz E., Kozma G.T., Pállinger É., Komlósi Z.I., Ádori C., Kovács L., Szebeni B., Rusai K., Losonczy G., Szabó A. (2009). Galectin-9 in Allergic Airway Inflammation and Hyper-Responsiveness in Mice. Int. Arch. Allergy Immunol..

[39] Sanchez-Cuellar S., de la Fuente H., Cruz-Adalia A., Lamana A., Cibrian D., Giron R.M., Vara A., Sanchez-Madrid F., Ancochea J. (2012). Reduced expression of galectin-1 and galectin-9 by leucocytes in asthma patients. Clin. Exp. Immunol..

[40] Katoh S., Nobumoto A., Matsumoto N., Matsumoto K., Ehara N., Niki T., Inada H., Nishi N., Yamauchi A., Fukushima K. (2010). Involvement of Galectin-9 in Lung Eosinophilia in Patients with Eosinophilic Pneumonia. Int. Arch. Allergy Immunol..

[41] Kubach J., Lutter P., Bopp T., Stoll S., Becker C., Huter E., Richter C., Weingarten P., Warger T., Knop J. (2007). Human CD4+CD25+ regulatory T cells: Proteome analysis identifies galectin-10 as a novel marker essential for their anergy and suppressive function. Blood.

[42] Melo R.C.N., Wang H., Silva T.P., Imoto Y., Fujieda S., Fukuchi M., Miyabe Y., Hirokawa M., Ueki S., Weller P.F. (2020). Galectin-10, the protein that forms Charcot-Leyden crystals, is not stored in granules but resides in the peripheral cytoplasm of human eosinophils. J. Leukoc. Biol..

[43] Persson E.K., Verstraete K., Heyndrickx I., Gevaert E., Aegerter H., Percier J.-M., Deswarte K., Verschueren K.H.G., Dansercoer A., Gras D. (2019). Protein crystallization promotes type 2 immunity and is reversible by antibody treatment. Science.

[44] Fu Y.-F., Jiang S.-C., Zhang Z.-W., Yang X.-Y., Li Z.-L., Hu J., Yuan S. (2023). Lactose and Galactose Promote the Crystallization of Human Galectin-10. Molecules.

[45] Na H., Sayed H., Ayala G.J., Wang X., Liu Y., Yu J., Liu T., Mayo K.H., Su J. (2023). Glutathione disrupts galectin-10 Charcot-Leyden crystal formation to possibly ameliorate eosinophil-based diseases such as asthma. Acta Biochim. Biophys. Sin..

[46] Yoshimura H., Takeda Y., Shirai Y., Yamamoto M., Nakatsubo D., Amiya S., Enomoto T., Hara R., Adachi Y., Edahiro R. (2024). Galectin-10 in serum extracellular vesicles reflects asthma pathophysiology. J. Allergy Clin. Immunol..

[47] Lingblom C., Andersson K., Wennerås C. (2020). Kinetic studies of galectin-10 release from eosinophils exposed to proliferating T cells. Clin. Exp. Immunol..

[48] Manna O.M., La Grutta S., Malizia V., Fucarino A., Rappa F., Picone D., Fasola S., Profita M., Bucchieri F., Gagliardo R. (2023). Role of Galectin-10 (Gal-10) in nasal epithelium inflammation and remodeling of children with seasonal allergic rhinitis (SAR). Eur. Respir. J..

[49] Virkud Y.V., Kelly R.S., Croteau-Chonka D.C., Celedón J.C., Dahlin A., Avila L., Raby B.A., Weiss S.T., Lasky-Su J.A. (2018). Novel eosinophilic gene expression networks associated with IgE in two distinct asthma populations. Clin. Exp. Allergy.

[50] Nyenhuis S.M., Alumkal P., Du J., Maybruck B.T., Vinicky M., Ackerman S.J. (2019). Charcot–Leyden crystal protein/galectin-10 is a surrogate biomarker of eosinophilic airway inflammation in asthma. Biomark. Med..

[51] Rodríguez-Alcázar J.F., Ataide M.A., Engels G., Schmitt-Mabmunyo C., Garbi N., Kastenmüller W., Latz E., Franklin B.S. (2019). Charcot–Leyden Crystals Activate the NLRP3 Inflammasome and Cause IL-1β Inflammation in Human Macrophages. J. Immunol..

[52] Kobayashi K., Nagase H., Sugimoto N., Yamamoto S., Tanaka A., Fukunaga K., Atsuta R., Tagaya E., Hojo M., Gon Y. (2021). Mepolizumab decreased the levels of serum galectin-10 and eosinophil cationic protein in asthma. Asia Pac. Allergy.

[53] Devouassoux G., Pachot A., Laforest L., Diasparra J., Freymond N., Van Ganse E., Mougin B., Pacheco Y. (2007). Galectin-10 mRNA is overexpressed in peripheral blood of aspirin-induced asthma. Allergy.

[54] Gevaert E., Delemarre T., De Volder J., Zhang N., Holtappels G., De Ruyck N., Persson E., Heyndrickx I., Verstraete K., Aegerter H. (2019). Charcot-Leyden crystals promote neutrophilic inflammation in patients with nasal polyposis. J. Allergy Clin. Immunol..

[55] Rabinovich G.A., Sotomayor C.E., Riera C.M., Bianco I., Correa S.G. (2000). Evidence of a Role for Galectin-1 in Acute Inflammation. Eur. J. Immunol..

[56] Perone M.J., Bertera S., Shufesky W.J., Divito S.J., Montecalvo A., Mathers A.R., Larregina A.T., Pang M., Seth N., Wucherpfennig K.W. (2009). Suppression of Autoimmune Diabetes by Soluble Galectin-1. J. Immunol..

[57] Yakushina V.D., Vasil’eva O.A., Ryazantseva N.V., Novitsky V.V., Tashireva L.A. (2014). The effects of galectin-1 on the gene expression of the transcription factors TBX21, GATA-3, FOXP3 and RORC. Mol. Cell. Biochem..

[58] Ge N.X., Gil Ha S., Greenberg Y.G., Rao A., Bastan I., Blidner A.G., Rao S.P., Rabinovich G.A., Sriramarao P. (2016). Regulation of eosinophilia and allergic airway inflammation by the glycan-binding protein galectin-1. Proc. Natl. Acad. Sci. USA.

[59] Pang X., Qiao J. (2020). Galectin-1 inhibits PDGF-BB-induced proliferation and migration of airway smooth muscle cells through the inactivation of PI3K/Akt signaling pathway. Biosci. Rep..

[60] Sewgobind N.V., Albers S., Pieters R.J. (2021). Functions and Inhibition of Galectin-7, an Emerging Target in Cellular Pathophysiology. Biomolecules.

[61] Tian J., He R., Fan Y., Zhang Q., Tian B., Zhou C., Liu C., Song M., Zhao S. (2020). Galectin-7 overexpression destroys airway epithelial barrier in transgenic mice. Integr. Zool..

[62] Sun X., Zhang W. (2018). Silencing of Gal-7 inhibits TGF-β1-induced apoptosis of human airway epithelial cells through JNK signaling pathway. Exp. Cell Res..

[63] Yi L., Zhang S., Feng Y., Wu W., Chang C., Chen D., Chen S., Zhao J., Zhen G. (2021). Increased epithelial galectin-13 expression associates with eosinophilic airway inflammation in asthma. Clin. Exp. Allergy.

[64] Ho M.K., Springer T.A. (1982). Mac-2, a novel 32,000 Mr mouse macrophage subpopulation-specific antigen defined by monoclonal antibodies. J. Immunol..

[65] Cooper D.N., Barondes S.H. (1999). God must love galectins; He made so many of them. Glycobiology.

[66] Fujisawa T., Velichko S., Thai P., Hung L.-Y., Huang F., Wu R. (2009). Regulation of Airway *MUC5AC* Expression by IL-1β and IL-17A; the NF-κB Paradigm. J. Immunol..

[67] Liu T., Zhang L., Joo D., Sun S.-C. (2017). NF-κB signaling in inflammation. Signal Transduct. Target. Ther..

[68] McKinley L., Alcorn J.F., Peterson A., DuPont R.B., Kapadia S., Logar A., Henry A., Irvin C.G., Piganelli J.D., Ray A. (2008). TH17 Cells Mediate Steroid-Resistant Airway Inflammation and Airway Hyperresponsiveness in Mice. J. Immunol..

[69] Liu F.-T., Stowell S.R. (2023). The role of galectins in immunity and infection. Nat. Rev. Immunol..

[70] Sato S., Ouellet N., Pelletier I., Simard M., Rancourt A., Bergeron M.G. (2002). Role of Galectin-3 as an Adhesion Molecule for Neutrophil Extravasation During Streptococcal Pneumonia. J. Immunol..

[71] Snarr B.D., St-Pierre G., Ralph B., Lehoux M., Sato Y., Rancourt A., Takazono T., Baistrocchi S.R., Corsini R., Cheng M.P. (2020). Galectin-3 enhances neutrophil motility and extravasation into the airways during Aspergillus fumigatus infection. PLOS Pathog..

[72] Rao S.P., Na Ge X., Sriramarao P. (2017). Regulation of Eosinophil Recruitment and Activation by Galectins in Allergic Asthma. Front. Med..

[73] Alexander W.S. (2002). Suppressors of cytokine signalling (SOCS) in the immune system. Nat. Rev. Immunol..

[74] López E., Zafra M.P., Sastre B., Gámez C., Lahoz C., del Pozo V. (2011). Gene Expression Profiling in Lungs of Chronic Asthmatic Mice Treated with Galectin-3: Downregulation of Inflammatory and Regulatory Genes. Mediat. Inflamm..

[75] Iwasaki-Hozumi H., Chagan-Yasutan H., Ashino Y., Hattori T. (2021). Blood Levels of Galectin-9, an Immuno-Regulating Molecule, Reflect the Severity for the Acute and Chronic Infectious Diseases. Biomolecules.

[76] Katoh S., Matsumoto N., Kawakita K., Tominaga A., Kincade P.W., Matsukura S. (2003). A Role for CD44 in an Antigen-Induced Murine Model of Pulmonary Eosinophilia. J. Clin. Investig..

[77] Inoue H., Fukuyama S., Matsumoto K., Kubo M., Yoshimura A. (2007). Role of endogenous inhibitors of cytokine signaling in allergic asthma. Curr. Med. Chem..

[78] Wiersma V.R., de Bruyn M., Helfrich W., Bremer E. (2011). Therapeutic potential of Galectin-9 in human disease. Med. Res. Rev..

[79] Su J. (2018). A Brief History of Charcot-Leyden Crystal Protein/Galectin-10 Research. Molecules.

[80] Melo R.C.N., Weller P.F. (2018). Contemporary understanding of the secretory granules in human eosinophils. J. Leukoc. Biol..

[81] Lao L.-M., Kumakiri M., Nakagawa K., Ishida H., Ishiguro K., Yanagihara M., Ueda K. (1998). The ultrastructural findings of Charcot-Leyden crystals in stroma of mastocytoma. J. Dermatol. Sci..

[82] Zhou Z., Teneri D.G., Dvorak A.M., Ackerman S.J. (1992). The gene for human eosinophil Charcot-Leyden crystal protein directs expression of lysophospholipase activity and spontaneous crystallization in transiently transfected COS cells. J. Leukoc. Biol..

[83] Dyer K.D., Rosenberg H.F. (1996). Eosinophil Charcot-Leyden crystal protein binds to beta-galactoside sugars. Life Sci..

[84] Yousefi S., A Gold J., Andina N., Lee J.J., Kelly A.M., Kozlowski E., Schmid I., Straumann A., Reichenbach J., Gleich G.J. (2008). Catapult-like release of mitochondrial DNA by eosinophils contributes to antibacterial defense. Nat. Med..

[85] Ueki S., Melo R.C.N., Ghiran I., Spencer L.A., Dvorak A.M., Weller P.F. (2013). Eosinophil extracellular DNA trap cell death mediates lytic release of free secretion-competent eosinophil granules in humans. Blood.

[86] Hashimoto T., Ueki S., Kamide Y., Miyabe Y., Fukuchi M., Yokoyama Y., Furukawa T., Azuma N., Oka N., Takeuchi H. (2022). Increased Circulating Cell-Free DNA in Eosinophilic Granulomatosis with Polyangiitis: Implications for Eosinophil Extracellular Traps and Immunothrombosis. Front. Immunol..

[87] De Re V., Simula M.P., Cannizzaro R., Pavan A., De Zorzi M.A., Toffoli G., Canzonieri V. (2009). Galectin-10, Eosinophils, and Celiac Disease. Ann. N. Y. Acad. Sci..

[88] Takeda M., Ueki S., Yamamoto Y., Nara M., Fukuchi M., Nakayama K., Omori Y., Takahashi N., Hirokawa M. (2020). Hypereosinophilic syndrome with abundant Charcot-Leyden crystals in spleen and lymph nodes. Asia Pac. Allergy.

[89] Smart C., Brown J., Kocjan G., Proctor I. (2011). Eosinophilic pleural effusion with Charcot–Leyden crystals in invasive aspergillosis. Cytopathology.

[90] Fukuchi M., Kamide Y., Ueki S., Miyabe Y., Konno Y., Oka N., Takeuchi H., Koyota S., Hirokawa M., Yamada T. (2021). Eosinophil ETosis–Mediated Release of Galectin-10 in Eosinophilic Granulomatosis with Polyangiitis. Arthritis Rheumatol..

[91] Farooque S.P., Lee T.H. (2009). Aspirin-Sensitive Respiratory Disease. Annu. Rev. Physiol..

[92] Dunican E.M., Elicker B.M., Gierada D.S., Nagle S.K., Schiebler M.L., Newell J.D., Raymond W.W., Lachowicz-Scroggins M.E., Di Maio S., Hoffman E.A. (2018). Mucus plugs in patients with asthma linked to eosinophilia and airflow obstruction. J. Clin. Investig..

[93] Aegerter H., Lambrecht B.N. (2023). The Pathology of Asthma: What Is Obstructing Our View?. Annu. Rev. Pathol. Mech. Dis..

[94] Aegerter H., Smole U., Heyndrickx I., Verstraete K., Savvides S.N., Hammad H., Lambrecht B.N. (2021). Charcot–Leyden crystals and other protein crystals driving type 2 immunity and allergy. Curr. Opin. Immunol..

[95] Linssen R.S., Chai G., Ma J., Kummarapurugu A.B., van Woensel J.B.M., Bem R.A., Kaler L., Duncan G.A., Zhou L., Rubin B.K. (2021). Neutrophil Extracellular Traps Increase Airway Mucus Viscoelasticity and Slow Mucus Particle Transit. Am. J. Respir. Cell Mol. Biol..

[96] Jackson D.J., Heaney L.G., Humbert M., Kent B.D., Shavit A., Hiljemark L., Olinger L., Cohen D., Menzies-Gow A., Korn S. (2024). Reduction of daily maintenance inhaled corticosteroids in patients with severe eosinophilic asthma treated with benralizumab (SHAMAL): A randomised, multicentre, open-label, phase 4 study. Lancet.

[97] Scioscia G., Nolasco S., Campisi R., Quarato C.M.I., Caruso C., Pelaia C., Portacci A., Crimi C. (2023). Switching Biological Therapies in Severe Asthma. Int. J. Mol. Sci..

[98] Campisi R., Nolasco S., Pelaia C., Impellizzeri P., D’amato M., Portacci A., Ricciardi L., Scioscia G., Crimi N., Scichilone N. (2023). Benralizumab Effectiveness in Severe Eosinophilic Asthma with Co-Presence of Bronchiectasis: A Real-World Multicentre Observational Study. J. Clin. Med..

